# Sarcoma of the Mediastinum of a Rhinoceros1Read before the Pathological Society of Philadelphia, February 14, 1901.

**Published:** 1901-03

**Authors:** Randle C. Rosenberger

**Affiliations:** Demonstrator of Bacteriology and Morbid Anatomy, Jefferson Medical College


### SARCOMA OF THE MEDIASTINUM OF A RHINOCEROS.[1]

By Randle C. Rosenberger, M.D.,[2]
DEMONSTRATOR OF BACTERIOLOGY AND MORBID ANATOMY, JEFFERSON MEDICAL COLLEGE.

(*From the Laboratories of the Jefferson Medical College.*)

The material upon which this report is based was obtained from an Indian rhinoceros that died in the Philadelphia Zoölogical Gardens. The animal was believed to be near a century old and was thought to have died from old age. There was no evidence of metastasis and the symptoms did not attract attention.

The specimen represents what is apparently a part of the pericardium, to which is attached a mass evidently of new formation. It consists of a slab of tissue measuring 30 cm. in length, 10 cm. in width, and 5 cm. in thickness. The incised surface is for the most part of a grayish or yellowish-white color; in some areas it is distinctly pinkish, in others dark brown. Upon one edge of the specimen is a comparatively smooth membrane 5 mm. in thickness, nearly pearly white in color, to which the adjacent mass is adherent. There are also seen in the mass proper numerous large

[1] Read before the Pathological Society of Philadelphia, February 14, 1901.

[2] The writer is indebted to Prof. H. C. Chapman for the privilege of examining and reporting this case.

and small processes of glistening white tissue coming off from the above-mentioned membrane, giving the surface an appearance resembling that seen on section of alveolar structures. In consistency the mass is moderately soft, in some areas nearly pultaceous, the finger readily sinking into the softened areas upon the slightest pressure. In one or two small areas upon the membrane above mentioned there is a thin, irregular layer of what appears to be fibrin, evidently a part of existing inflammation.

Small masses were taken for histological examination. These were fixed in absolute alcohol and embedded in paraffin. The specimen as a whole was preserved by Kaiserling's method. Sections were cut and stained with hæmatoxylin alone, hæmatoxylin and eosin, hæmatoxylin and picric acid, toluidin-blue and eosin, carbol-fuchsin and methylene-blue.

Histological examination shows the specimen to be made up for the most part of small round-cells. Beside the cellular elements just mentioned there is a quantity of fibrous connective tissue which extends in a quite lawless manner between the round-cell elements. From these fibrous trabeculæ arise numerous more or less spindle-shaped cells that are distributed between the small round-cells.

Numerous large and small bloodvessels are seen throughout the sections; they are distributed irregularly and many of them possess no definite walls, being nothing more than sinuses limited by masses of the tumor cells. In other areas the bloodvessels are situated in the fibrous connective tissue; in this location infiltration of the vascular walls by round-cells of the mass proper is clearly shown. Most of the vessels are empty, although a few contain erythrocytes and leukocytes. In those sections stained with toluidin-blue and eosin mast-cells or mucinoblasts are seen in the connective tissue. The areas upon the membrane that were thought to be fibrinous are seen to be made up of fibroblasts and small round-cells. No cancer parasites were demonstrable. Pigment is present, being found in the tumor cells and in the connective tissue; it consists of small brownish-yellow granules, occasionally massed in groups approximating in size the volume of adjacent cells.

Diagnosis: Sarcoma, the predominating element of which is a small round-cell; the quantity of pigment is hardly sufficient to justify our calling the neoplasm melanotic.

